# 2315. Hidden in Plain Sight: Latent Class Analysis of Respiratory Infection Symptoms based on COVID-19 Testing and Vaccination History

**DOI:** 10.1093/ofid/ofad500.1937

**Published:** 2023-11-27

**Authors:** Xiaowu Sun, Laura L Lupton, Coetzer Henriette, Jonathan DeShazo, Alexandra Berk, Laura Anatale-Tardiff

**Affiliations:** CVS Health, Woonsocket, Rhode Island; CVS Health Clinical Trial Services, Woonsocket, Rhode Island; CVS Health, Woonsocket, Rhode Island; CVS Health, Woonsocket, Rhode Island; CVS Health Clinical Trial Services, Woonsocket, Rhode Island; CVS Health Clinical Trial Services, Woonsocket, Rhode Island

## Abstract

**Background:**

During XBB variant dominance, there is still a lack of evidence describing phenotypes of acute respiratory infection (ARI) symptoms present at COVID testing and how clusters of symptoms are associated with COVID test positivity, COVID vaccination status, and vaccination timing.

**Figure:**
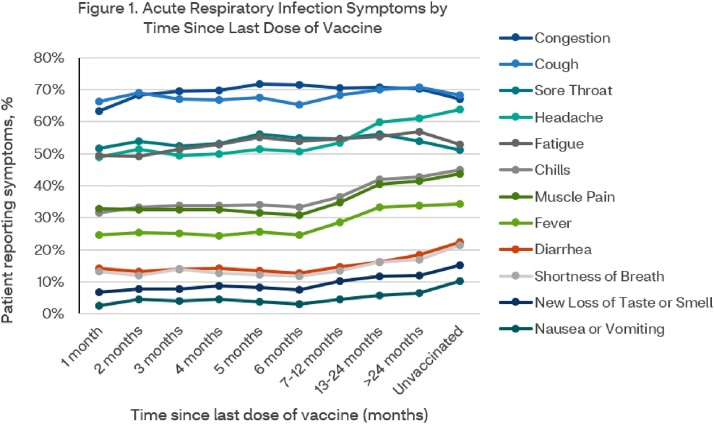

**Figure:**
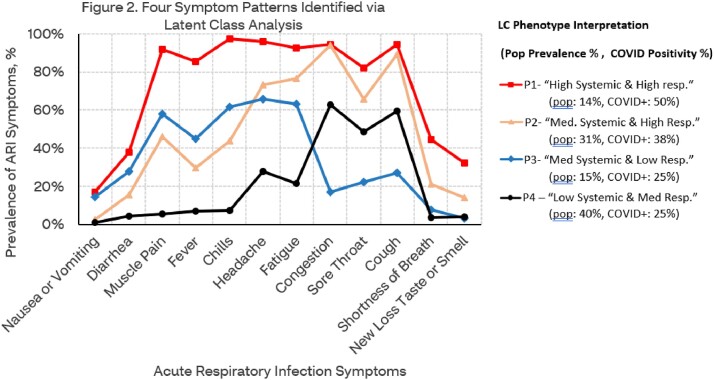

**Methods:**

This observational cohort included 88,147 adult symptomatic individuals presenting for COVID testing at CVS Pharmacy® locations across the US during March and April of 2023. Individuals self-reported ARI symptoms, comorbid conditions, vaccination status, vaccination timing, and demographics when making an appointment for COVID testing. Latent class analysis (LCA) is a statistically robust method of cluster analysis and was adopted to examine patterns of ARI symptoms. COVID testing positivity was tabulated for each pattern by time from last dose of vaccine.

**Results:**

Patients were 43.3 (SD: 16.6) years old on average and 37% male. The cohort was composed of 43% White or Caucasian, 21% Hispanic, 16% Black, 11% Asian, and 9% other race and/or ethnicities. About 20% of patients self-reported at least one comorbid condition. PCR and antigen tests were chosen by 79% and 21% of patients, respectively. Overall, 33% of patients tested positive for COVID. Of 87% vaccinated, 4% had been vaccinated within three months of testing, 21% within 4-6 months, 17% within 7-12 months, and 45% had been vaccinated over one year prior to testing.

The number of months since vaccination was associated with an increased number of reported symptoms, presenting a marked uptick of symptoms at 6-months following the last vaccination dose (Figure 1). LCA identified four distinct phenotype groups of ARI symptoms described with prevalence (%) (Figure 2). Phenotype group positivity rates were also associated with time since last dose of vaccines, and yet unlike individual symptoms, appeared to stabilize six months post-vaccination.

**Conclusion:**

Four ARI symptom phenotypes having distinct responses to vaccine and COVID positivity were identified. Some phenotypes (such as P3 and P4) may present with symptom patterns consistent with seasonal influenza or RSV. More research is needed to validate these findings and understand the implications of possible COVID phenotypes.

**Disclosures:**

**Xiaowu Sun, PhD**, CVS Health: Employee|CVS Health: Stocks/Bonds **Laura L. Lupton, MD, MHSA**, CVS Health: employee|CVS Health: Stocks/Bonds **Coetzer Henriette, MD**, Astra Zeneca: Stocks/Bonds|CVS Health: Employee|GSK: Stocks/Bonds **Alexandra Berk, PhD**, CVS Health: employee|CVS Health: Stocks/Bonds **Laura Anatale-Tardiff, MPH**, CVS Health: employee|CVS Health: Stocks/Bonds

